# Bioinformatics analysis reveals a stem cell-expressed circ-Serpine2-mediated miRNA-mRNA regulatory subnetwork in the malignant progression of glioma

**DOI:** 10.1186/s12967-021-03118-4

**Published:** 2021-10-24

**Authors:** Guowei Li, Qing Lan

**Affiliations:** Department of Neurosurgery, The Second Affiliated Hospital of Soochow University, Suzhou, China

**Keywords:** Glioma stem cell, circRNAs, Bioinformatic analysis, Malignant progression, Nexus

## Abstract

**Background:**

High-grade glioma has a poor prognosis, and GSCs can have pivotal roles in glioma pathology. This study investigated GSC exosome-containing circRNA mechanisms affecting the malignant progression of glioma.

**Methods:**

In this study, we identified differentially expressed circRNAs and constructed a circRNA-miRNA-mRNA regulatory network through circRNA sequencing/bioinformatics analysis. Then, we identified circRNAs that were upregulated in GSC23 cells and employed them as downstream targets in subsequent investigations. Such investigations included downstream target knockout to assess any influence on A172 cell proliferation, invasion, migration and apoptosis. In addition, in vivo investigations using tumor-bearing animals evaluated the in vivo influences of the selected targets.

**Results:**

This study identified circ-Serpine2/miR-124-3p/KIF20A as a regulatory pathway in glioma. Our in vitro analysis confirmed that circ-Serpine2 could upregulate KIF20A by sponging miR-124-3p, consequently promoting A172 cell proliferation, migration and invasion. Such a signaling channel could also inhibit glioma cell apoptosis. Additionally, our research indicated that circ-Serpine2 inhibited glioma apoptosis and promoted in vivo tumor progression.

**Conclusion:**

Circ-Serpine2 exacerbated the malignant progression of glioma mediated by the miR-124-3p/KIF20A nexus, thus providing novel predictive/prognostic biomarkers and drug targets against glioma.

## Introduction

High-grade glioma (WHO III-IV) is a malignant tumor that possesses reduced prognostic profiles, although glioma therapy advancements have been established. Approximately 5 in 100,000 people are newly diagnosed with glioma annually, and glioblastomas represent approximately 15% of all primary brain tumors [1–9]. Currently, surgical resection followed by concomitant radiochemotherapy is considered to be an efficient treatment. However, most of these therapies have not led to increased survival rates, and such grim realities force us to seek new treatments.

Local recurrences contribute to the poor outcomes of glioma. Increasing evidence suggests that these pathological processes are caused by tumor-initiating glioma stem cells (GSCs), and it has been documented that exosomes released by GSCs might play important roles in glioma progression [10–15]. Therefore, it is necessary to investigate the stem cell-related mechanisms that underlie glioma development and identify potential therapeutic targets.

In recent decades, rising quantities of cancer-related circRNAs have been observed, and several of them were found to take up oncogenic or tumor-suppressive activities within human malignancies. Multiple investigations have suggested that circRNAs induce cytoplasmic downregulation of targeted microRNAs (miRNAs), consequently liberating downstream target transcripts of such downregulated miRNAs [16–18]. This study merged a previously developed circRNA database with the Gene Expression Omnibus (GEO) database to explore circRNA-miRNA-mRNA networks in glioma. Additionally, founded upon upregulated expression circRNA profiles in GSCs, this study developed a network of regulating molecular members that consisted of eight circRNAs, six miRNAs, and ten mRNAs using the GEO database and additional similar repositories for predictive purposes. Gene ontology (GO) annotation, Encyclopedia of Genes and Genomes (KEGG) pathway analyses and a protein–protein interaction (PPI) network were used to unravel putative regulatory mechanisms of circRNAs in the progression of glioma. Finally, we filtered out the signaling pathway (circ-Serpine2/miRNA-124–3p/KIF20A) and clarified the regulatory mechanism through expression validation and functional verification, rendering such molecular players highly appealing as novel drug targets.

## Materials and methods

### Cell culture

The GSC23 line was a gift from the Soochow University Stem Cell Research Group, and these cells were cultured in serum-free medium (Gibco, USA). Then, we added serum to the medium to induce GSC23 differentiation into glioma cells. A172, U251 and SVG cells were procured from Shanghai Institutes for Biological Sciences and grown in RPMI 1640 medium (Gibco, USA) supplemented with 10% fetal bovine serum (FBS) (Gibco, USA), 100 ng/mL streptomycin, and 100 U/mL penicillin (Gibco, USA). All cultures incubated at 37 °C and 5% CO_2_.

### Exosome isolation and identification

Exosomes were extracted from cell culture supernatants by ultrahigh-speed centrifugation. Briefly, cell culture supernatants were differentially centrifuged (300*g* for 10 min, 1000*g* for 20 min, or 10,000*g* for 30 min). After filtration, the supernatant was ultracentrifuged at 100,000*g* for 180 min. Then, the precipitates were subjected to two phosphate-buffered saline (PBS)-washing steps, resuspended in PBS and stored at − 80 °C. Exosomes were visualized by scanning electron microscopy and confirmed by protein concentration determination, and the expression levels of CD9, CD63, Annexin, and Calnexin were assessed using Western blotting.

### Scanning electron microscopic observation and measurement

Exosomal morphologies were visually assessed by scanning electron microscopy. The GSC23 and glioma cell supernatants were collected and extracted by ultrahigh-speed centrifugation, and a small amount of white sediment was observed at the bottom of the tube after centrifugation. Then, it was dissolved in 1 × PBS, fixed with 2.5% glutaraldehyde and dehydrated. Finally, we used a scanning electron microscope for observation. The samples were diluted with PBS, and 100 μL dilution was added to the sample plate. An Izon particle analyzer and a high-resolution tunable resistance pulse were used to detect the particle diameter.

### CircRNA sequencing

Total RNA was extracted from GSC23/GSC23-differentiated cells with TRIzol (Life Technologies, ™, Carlsbad, CA, USA). Before constructing RNA-seq libraries, both the Ribo-Zero rRNA Removal Kit (Illumina, San Diego, CA, USA) and CircRNA Enrichment Kit (Cloud-seq, USA) were employed to attain rRNA removal and circRNA enrichment. The RNA-seq libraries were developed through pretreatment of RNAs with the TruSeq® Stranded Total RNA Library Prep Kit (Illumina,™, San Diego, CA, USA). All libraries were denatured into single-stranded DNA molecules, placed onto flow cells, amplified in situ as clusters and consequently sequenced (150 cycles) on an Illumina HiSeq™® 4000 Sequencer (Illumina,™, San Diego, CA, USA).

### Microarray data and RNA sequencing data

Microarray datasets were retrieved from the GEO database. CircRNA sequencing was conducted in the Experimental Center of the Second Affiliated Hospital of Soochow University.

### Expression profile assessments

CircRNA expression profiling for GSC23- and GSC23-differentiated cells (3 replicates each) was obtained by circRNA microarray technology. Cell circRNA expression difference analysis was performed using the limma package in R, and |logFC|> 1 and P < 0.05 were considered statistically significant.

According to gene ceRNA theory, highly expressed circRNAs may release miRNAs from their target mRNAs, thus promoting mRNA expression. Therefore, specifically overexpressed circRNAs in GSCs may act on miRNAs in glioma and result in negative regulation, thereby indirectly promoting abnormally high mRNA expression in glioma cells. To assess the interactions between circRNAs in GSCs and miRNAs in glioma cells, we first used the GEOquery package in R to determine the miRNA expression profile in the GSE25632 dataset in the GEO database (5 normal controls and 82 glioma tissues). The miRNA expression profile was annotated through the GPL8179 platform, and differential analysis was performed with the limma package in R. |logFC|> 1 and P < 0.05 were considered statistically significant.

To further explore the mRNA regulated by miRNA in glioma cells and specifically highly expressed circRNAs in GSCs, we used the GEOquery package in R to determine the mRNA expression profile from the GSE103227 dataset in the GEO database (including 5 normal controls and 5 glioma tissues). Differential analysis was performed with the limma package in R, with |logFC|> 1 and P < 0.05 being considered statistically significant.

### CircRNA-miRNA-mRNA regulatory network development

We used the miRNA-circRNA module on the StarBase v3.0 website (http://starbase.sysu.edu.cn) to predict the targeted relationship between the miRNAs and the specifically highly expressed circRNAs in GSCs. Then, a Venn diagram was used to determine the intersection between miRNAs specifically targeting highly expressed circRNAs in GSCs and miRNAs that are specifically expressed at low levels in glioma cells. Thus, we identified target miRNAs and used Cytoscape 3.8.2 to draw the circRNA-miRNA regulatory network of specifically highly expressed circRNAs in GSCs that may participate in glioma gene regulation.

We used the miRTarBase website (http://mirtarbase.mbc.nctu.edu.tw/php/index.php) for target correlation prediction between miRNAs and mRNAs in a regulatory network of circRNAs that are highly expressed in GSCs and glioma miRNAs. Then, a Venn diagram was used to determine the intersection between mRNAs specifically targeting miRNAs with low expression in glioma cells and mRNAs that are specifically highly expressed in glioma cells; thus, we identified target mRNAs and used Cytoscape 3.8.2 to draw the miRNA-mRNA regulatory network of specifically highly expressed circRNAs in GSCs that may participate in glioma gene regulation.

The circRNA-miRNA-mRNA regulatory network was developed by combining circRNA-miRNA/miRNA-mRNA pairs. Ultimately, network visualization was obtained through Cytoscape 3.8.2.

### Functional enrichment analysis

The R-based ‘Cluster Profiler package’ was employed for GO/KEGG pathway enrichment analysis of mRNA in the miRNA-mRNA regulatory network, followed by visualization of the results through Cytoscape 3.8.2. In addition, all such GO/KEGG outcomes were collected using R studio/R scripting language, and placed a criterion that GO analysis-P value would be < 0.05, and the P and Q values of KEGG analysis would be < 0.05.

### Construction of the PPI network, module and immune-correlation analysis

We used CGGA data and Kaplan–Meier survival analysis to delve into deeper assessment of the interactions of mRNA expression levels and the prognosis of glioma patients. Consequently, glioma poor prognosis-related mRNAs were submitted to the STRING version 11.0 (https://string-db.org/) database to develop the PPI network, species were restricted to Homo sapiens with medium confidence > 0.4, and isolated targets were removed. Then, the PPI network map was imported into Cytoscape 3.8.2 software for visualization, and the top 10 hub genes were analyzed by means of the cytoHubba plug-in. We further combined the glioma data in the GTEx and TCGA databases to compare expression level profiles for these 10 hub genes (glioma and healthy tissue). The Molecular Complex Detection (MCODE) app was employed for screening hub gene modules within this PPI network. Then, we used the GSVA R package to analyze the relationship between these 10 mRNAs and the degree of immune cell infiltration.

### Quantitative reverse transcription polymerase chain reaction (qRT-PCR)

Total RNA was isolated with TRIzol® (Invitrogen™, USA). The quality of total RNA was assessed using a NanoDrop® 1000 Spectrophotometer (Thermo Fisher Scientific™, USA). First-strand cDNA was prepared using the QuantiTect® Reverse Transcription Kit (QIAGEN™, USA). Actual RT-qPCR runs were conducted through qPCR SYBR Green Mix® (Bio-Rad™, USA) and the ABI 7500® platform (Applied Biosystems™, USA). PCRs were conducted using three technical replicates/sample, with resulting data assessed through the 2^−ΔΔCt^ methodology [18]. GAPDH served as a normalizing/reference gene for circRNA, mRNA and circRNA, while U6 served as a reference for circRNA. Primers consisted of GAPDH: 5′-AGAAGGCTGGGGCT CATTTG-3′ (forward) and 5′-AGGGGCCATCCACAG TCTTC-3′ (reverse); U6: 5′-CTCGCTTCGGCAGCACA-3′ (forward) and 5′-AACGCTTCACGAATTTGCG T-3′ (reverse).

### Cell treatment and transfection

SiRNAs targeting circRNA-Serpine2, miR-124-3p/KIF20A mimics and inhibitors were procured through GenePharma (JiangSu, China). Such oligonucleotides/vectors underwent transient transfection using Lipofectamine 3000® (Invitrogen™, USA) following the manufacturer's protocols. Regarding circRNA-Serpine2 knockdown, GSC23 cellular aliquots were transfected with 20 nM siRNA against circRNA-Serpine2. MiR-124-3p/KIF20A overexpression or knockdown was achieved with 20 nM miR-124-3p/KIF20A mimic or inhibitor in A172 cells. GSC23, A172 and U251 cells underwent transduction for 24 h. Consequently, qPCR was conducted to validate altered circRNA/miRNA/mRNA expression within stabilized cells.

### Western blot

Cellular lysis was conducted using RIPA® buffer (CWBio™, Beijing, China). Lysates were consequently placed into loading buffer and denatured (100 °C for 10 min). The resulting solutions were exposed to sodium dodecyl sulfate–polyacrylamide gel electrophoresis (SDS-PAGE), followed by transfer onto polyvinylidene difluoride (PVDF) membranes (Millipore™, Billerica, Massachusetts, USA). Following a 60-min blocking step, the membranes were placed into an incubator overnight at 4 °C in tandem with selected primary antibodies (Abcam™, Cambridge, MA, USA). Consequently, the relevant secondary antibody (CST™, Danvers, MA, USA) was introduced, and the membrane was kept at room temperature for 120 min. Proteomic loads were assessed using Immobilon Western Chemiluminescent HRP Substrate® (Millipore™, USA). The GAPDH signal was used as a loading control.

### Cell proliferation assay

For clone formation assays, transfected cells were seeded within 6-well plates and grown in medium with 10% FBS at 37 °C/5% CO_2_. Following a 10-day incubation period, cells were stained using 0.1% crystal violet (Beyotime™, Beijing, China), followed by manual colony counting.

### Cell invasion assay

Cell invasion assays were carried out using 24-well Transwells (8 μm, Corning, USA) coated with Matrigel (BD, USA). A172 cells were grown within circ-siRNAs, miRNA inhibitors and mRNA mimics according to the manufacturer’s instructions. Overall, 1 × 10^5^ cells in 500 μL DMEM (1% FBS) were added to the upper chamber, and 750 μL DMEM (10% FBS) was added to the lower chamber. After incubation for 48 h, Matrigel and cells in the upper chamber were removed. Cells on the lower surface of the membrane were fixed in 4% paraformaldehyde and stained with 0.5% crystal violet. The invasive cells were imaged using an inverted microscope (Nikon, Japan) and quantified in five random fields per well. Each trial had three independent experiments.

### Cell migration assay

A172 cells were grown within 6‐well plates together with circ-siRNAs, miRNA inhibitors or mRNA mimics according to the manufacturer’s instructions. Seventy-two hours later, serum-free DMEM was introduced to the plates for 12 h. All cells were seeded in marked plates and subjected to three consecutive wash steps using sterile PBS, and monolayers were scratched (plate central region) using 200-μL pipettor tips. After incubation under normal conditions for 24 h, the cells were evaluated microscopically. All assays were performed on three separate occasions.

### Apoptosis assay

A172 cells were harvested, washed in PBS and incubated with an Annexin V-FITC Apoptosis Detection Kit® (Beyotime Biotech™, Haimen, China) 48 h after transfection. Annexin V-FITC acted as a cell-staining agent, followed by resuspension into binding buffer (190 μL) prior to the introduction of 10 μL of PI (20 μg/mL). Consequently, the cells were incubated (15 min/dark conditions/RT) and then assessed through flow cytometry and FACSDiva® software (Version 6.2). Cell types were separated/grouped into viable, necrotized, and apoptotic cells, and the percentages of apoptotic cells within individual groups were determined.

### Xenograft study

Ten BALB/c nude murines (with 200 μL of cell suspension per mouse, 21 days old) were segregated in a random manner into two groups. In the control group, A172 cells were subcutaneously introduced within the right anterior flank of each mouse continuously for a five-day period. Concomitantly, circ-Serpine2 knockout exosomes were collected and injected across subcutaneous tumor circumferences at 0, 6, 12, 18, and 24 days post A172 injection. In the experimental group, mice were subcutaneously treated with A172 cells and GSC23 exosomes as described above. Day 6, 12, 18, 24 and 30 postsubcutaneous injection of A172 cells served as assessment time points, where tumor size was measured through calipers, and tumor volume (mm^3^) was assessed accordingly: length × width^2^/2.

Thirty days posttreatment, all mice were euthanized (pentobarbital sodium by intraperitoneal injection, 150–200 mg/kg). Murine tumors were removed and weighed, with sections collected, hematoxylin/eosin stained and placed for observation under microscopy measures.

### Statistical analysis

Statistical analysis was conducted using SPSS 21.0® (Chicago, IL, USA). Major circRNA dysregulations were assessed through R. Limma packages/FDR filtering were employed for comparative analyses. A P-value < 0.05 and absolute fold change ≥ 2 were deemed to confer statistical significance. The chi-square test was used to assess the interactions of mRNA expression level and clinical characteristics.

## Results

### Discovery of specifically expressed circRNAs in GSC23

**Fig. 1:**
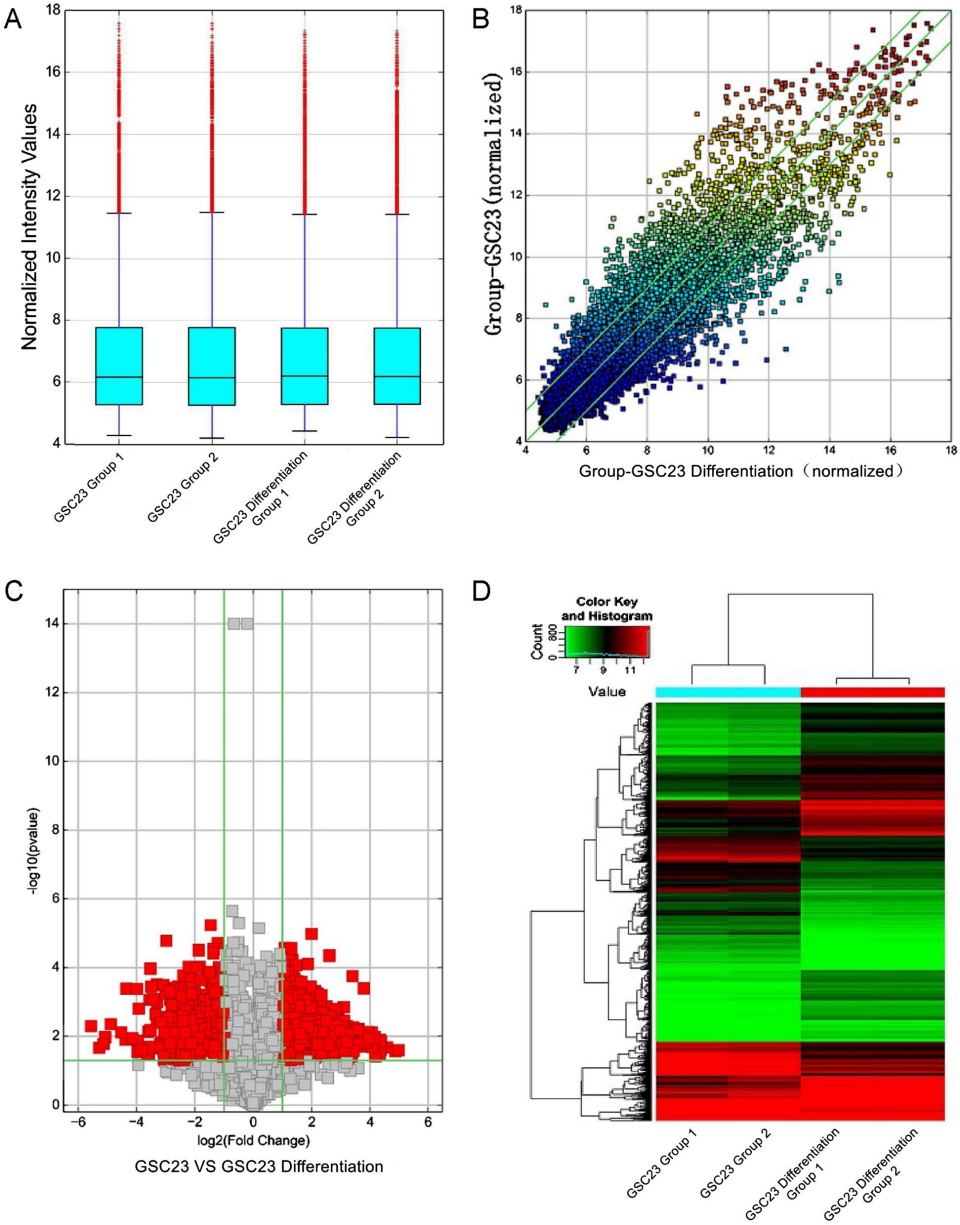
CircRNA sequencing of GSC23 and GSC23-differentiated cell-secreted exosomes. **A** Boxplot of GSC23 and differentiated GSC23 circRNA-seq. **B** Scatter plot of GSC23 and differentiated GSC23 cell circRNA-seq. **C** Volcano plot of circRNA-seq, upregulated (right side red), downregulated (left side red) or unchanged (gray). **D** Hierarchical cluster analysis revealed significantly upregulated/downregulated circRNAs between GSC23- and differentiated GSC23

**Fig. 2:**
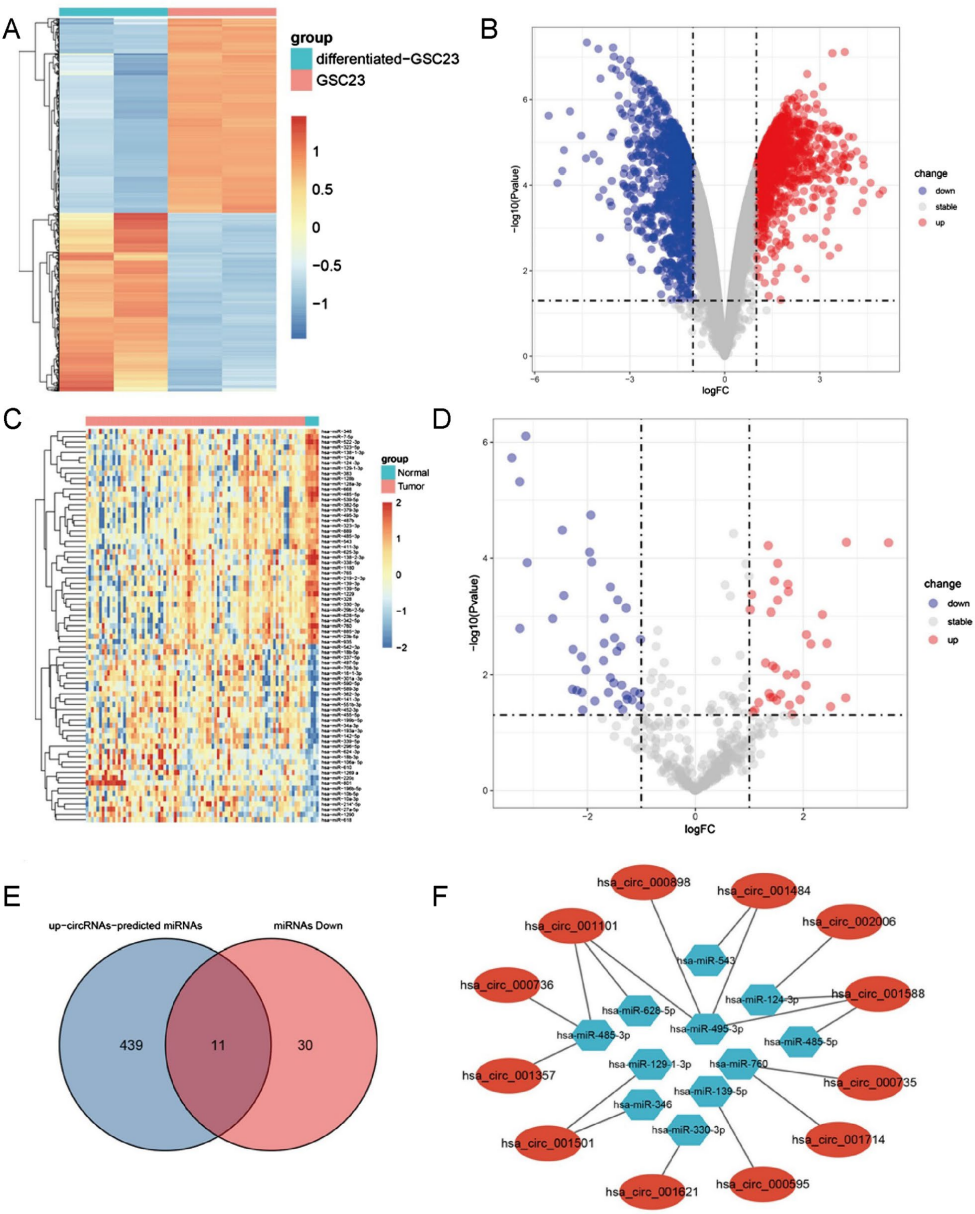
Discovery of circRNAs specifically expressed in GSCs and construction of a GSC-specifically expressed circRNA-miRNA regulatory network. **A**, **B** Abnormal circRNA expression in GSC23 was demonstrated in the heat map and volcano plot. **C**, **D** The dysregulated miRNA expression profile in glioma cells is demonstrated in the heat map and volcano plot. **E** We screened out 11 target miRNAs. **F** Cytoscape 3.8.2 was used to draw the GSC23-highly expressed circRNA-miRNA regulatory networks

CircRNAs in GSC23 and GSC23-differentiated cell-secreted exosomes were sequenced, and scatter, box, and volcano plots, as well as hierarchical clustering, were used to visualize differential expression patterns (Fig. 1). Candidate upregulated circRNAs were analyzed using primer screening and RT-PCR. Through microarray technology and the limma package in R, we identified differentially expressed circRNAs, and the results showed that compared with differentiated GSC23 cells, GSC23 cells had 2270 abnormally expressed circRNAs (1182 significantly upregulated; 1088 significantly downregulated). These abnormally expressed circRNAs were visualized in the form of heatmaps and volcano plots (Fig. 2A, B).

### Construction of the GSC23-glioma-specific circRNA-miRNA regulatory network

In the analysis, a total of 75 abnormally expressed miRNAs were identified (34 upregulated; 41 downregulated). We visualized the abnormally expressed miRNAs in glioma cells with heatmaps and volcano plots (Fig. 2C, D). Then, we preliminarily screened 450 miRNAs and selected 11 miRNAs from among them (i.e., hsa-miR-124-3p, hsa-miR-129-1-3p, hsa-miR-139-5p, hsa-miR-139-5p, hsa-miR-139-5p, hsa-miR-139-5p, hsa-miR-330-3p, hsa-miR-346, hsa-miR-485-3p, hsa-miR-485-5p, hsa-miR-543, hsa-miR-628-5p, hsa-miR-330-3p, hsa-miR-346, hsa-miR-760) (Fig. 2E). Finally, we used Cytoscape 3.8.2 to draw the regulatory networks of highly expressed circRNAs-miRNAs in GSC23 (Fig. 2F).

### Construction of the GSC23-glioma-specific miRNA-mRNA regulatory network

**Fig. 3:**
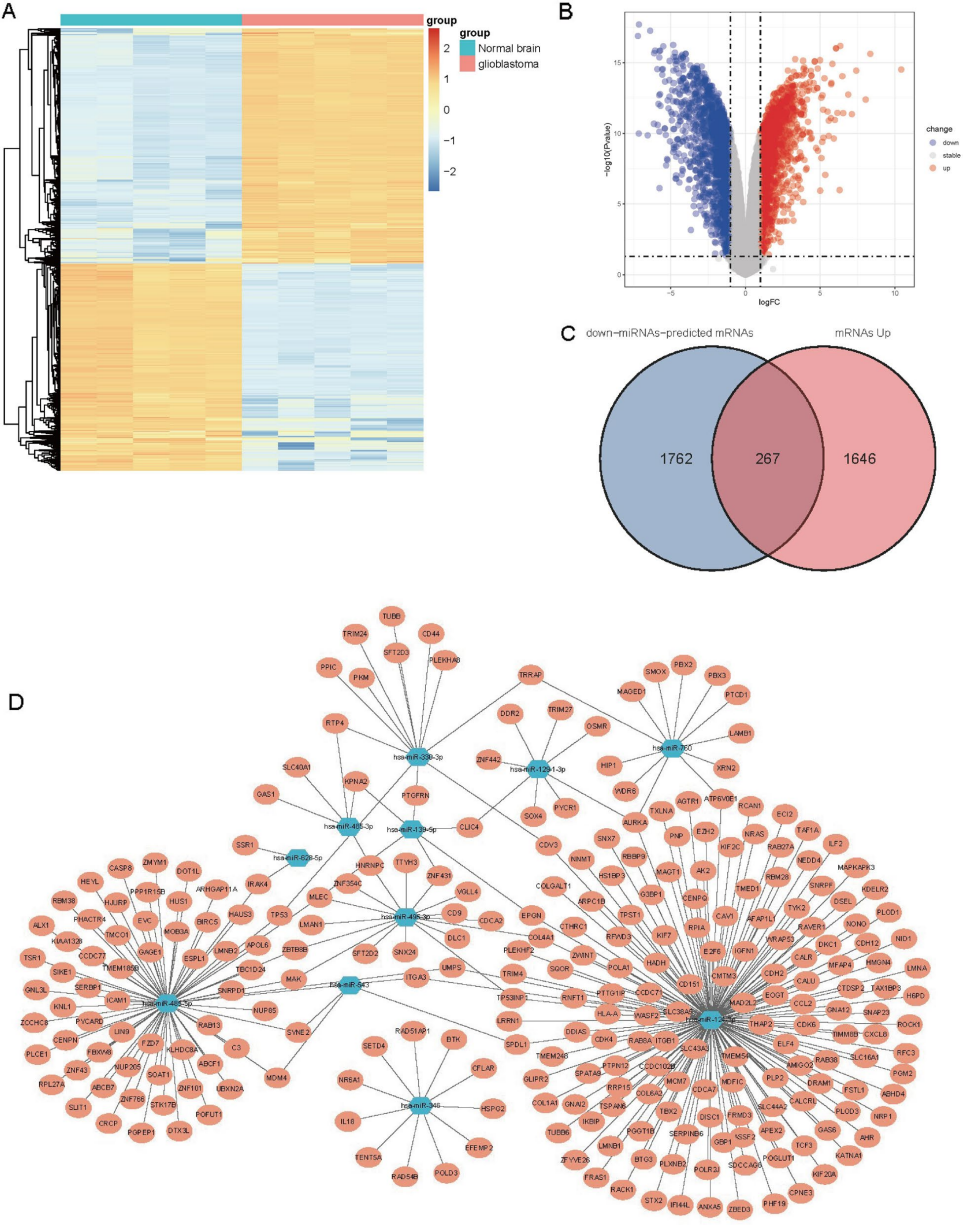
Construction of a GSC23-specifically expressed miRNA-mRNA regulatory network. **A**, **B** The abnormal expression of mRNA in glioma cells is demonstrated in the heat map and volcano plot. **C** In total, 267 target mRNAs were screened out. **D** Cytoscape 3.8.2 was used to draw the miRNA-mRNA regulatory network associated with highly expressed circRNAs in GSC23 that may be involved in the progression of glioma

We identified 3600 abnormally expressed mRNAs (1913 upregulated; 1687 downregulated). Abnormally expressed mRNAs in glioma cells were visualized by means of heatmaps and volcano plots (Fig. 3A, B). Then, we screened a total of 267 target mRNAs (Fig. 3C). Finally, we used Cytoscape 3.8.2 to draw the GSC23-glioma-specific miRNA-mRNA regulatory network that may be involved in glioma gene regulation (Fig. 3D).

### Functional exploration of mRNAs in the GSC23-specific miRNA-mRNA regulatory network

**Fig. 4:**
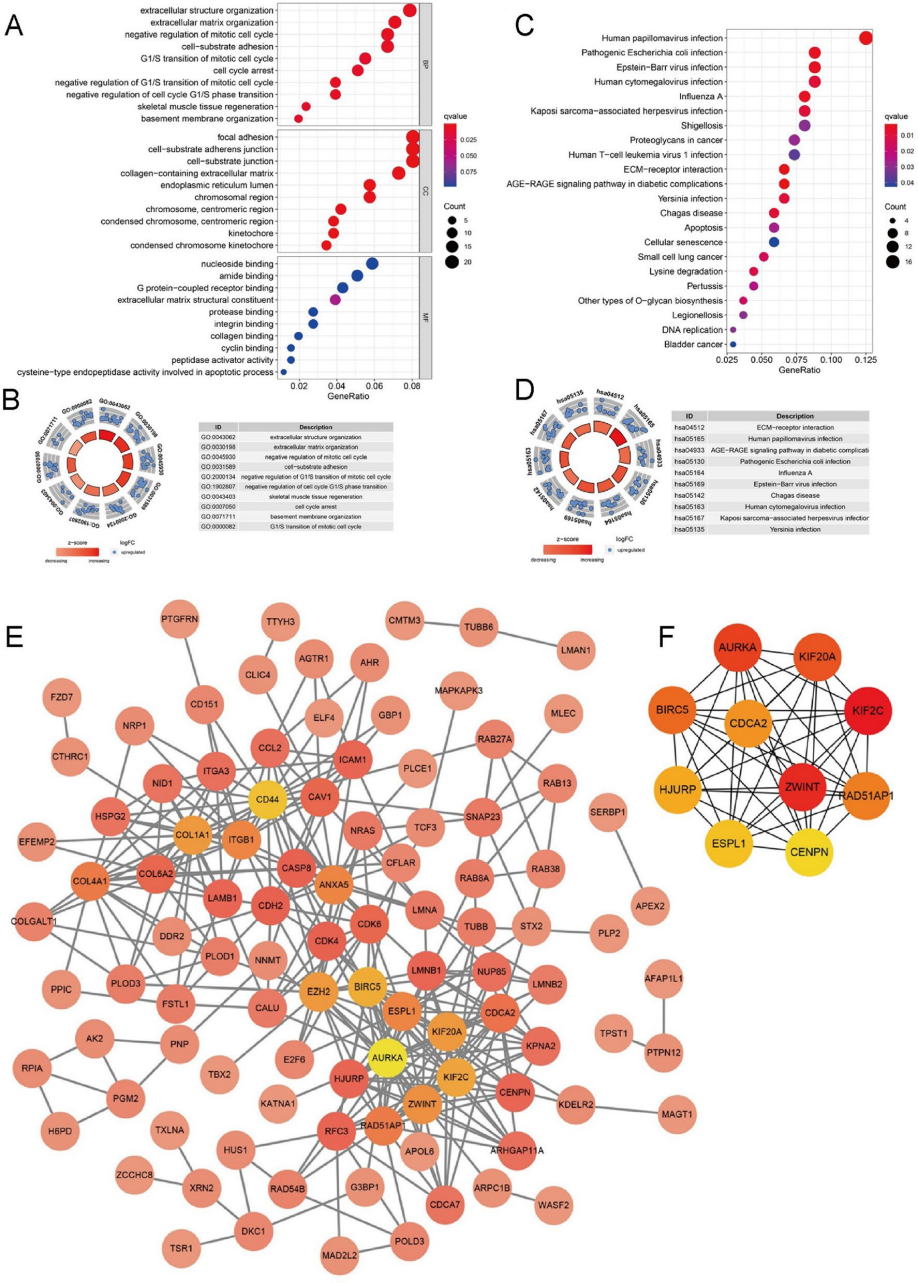
Functional exploration of mRNAs in the GSC-specifically expressed miRNA-mRNA regulatory network. **A**, **B** GO analysis revealed the main biological processes and molecular functions that were associated with these 267 mRNAs. **C**, **D** KEGG pathway enrichment analysis results suggested that these 267 mRNAs were highly enriched within cancer-related pathways. **E** The PPI network diagram was imported into Cytoscape3.8.2 software for visualization. **F** The 10 top-ranking hub genes were flagged through cytoHubba analysis

GO analysis demonstrated 267 mRNAs to be predominantly correlated with extracellular structure organization, extracellular matrix organization, deregulation of mitotic cell cycle and other biological processes, focal adhesion, cell-substrate adherens junction, cell-substrate junction, nucleoside binding, amide binding, G protein-coupled receptor binding and other molecular functions (Fig. 4A, B). KEGG pathway-enrichment analysis results suggested that these 267 mRNAs were significantly enriched within cancer-related pathways, such as ECM-receptor interaction, apoptosis and DNA replication (Fig. 4C, D). These results suggested that circRNAs specifically expressed in GSC23 may regulate these pathways through miRNA-mRNA networks, thus affecting glioma progression.

### Construction of the GSC23-glioma-unique circRNA-miRNA-mRNA regulatory network

**Fig. 5:**
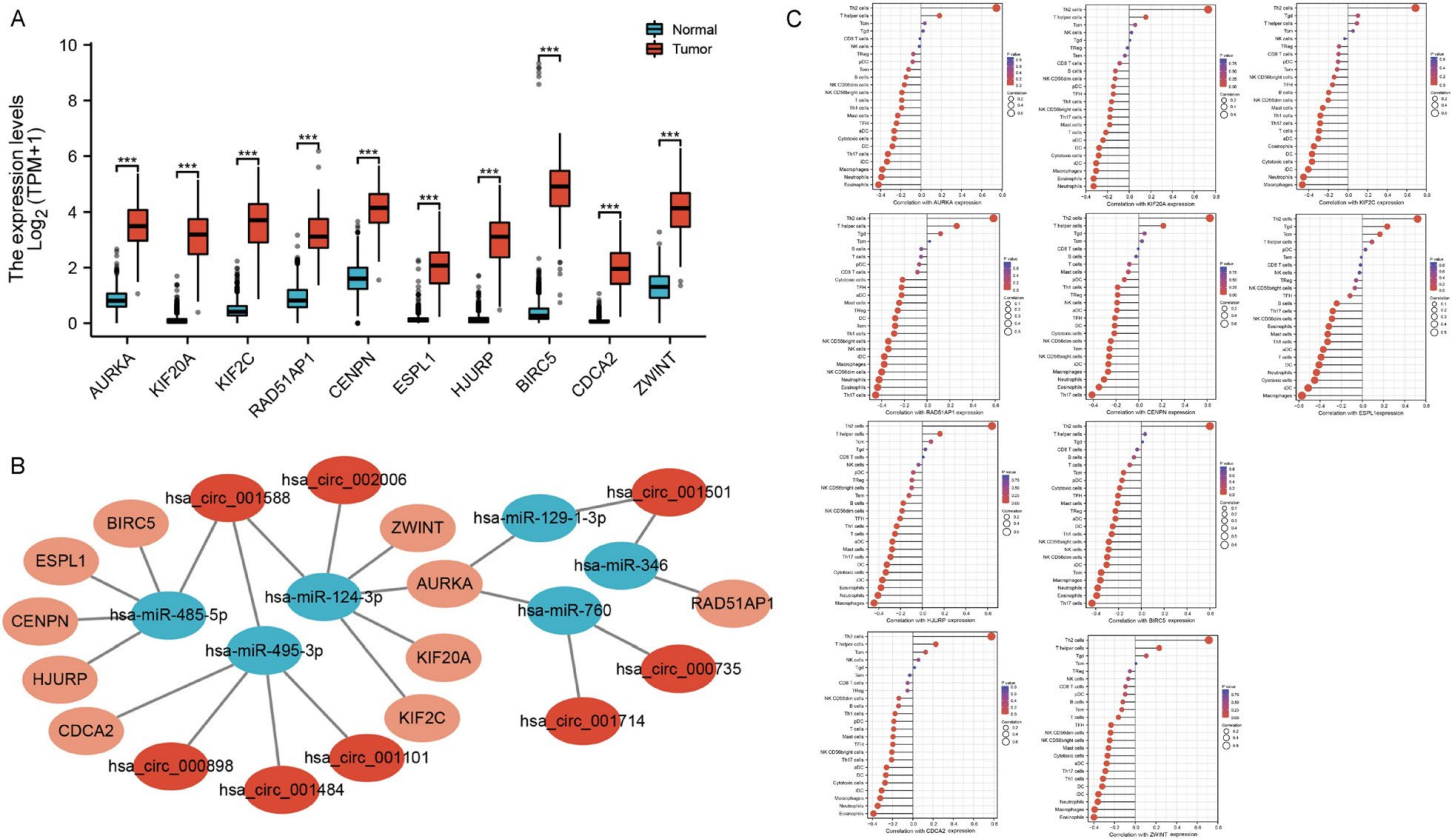
Development of a GSC23-specifically expressed circRNA-miRNA-mRNA network and immune-correlation analysis. **A** We found that 10 hub genes in glioma tissue were upregulated. **B** A circRNA-miRNA-mRNA regulatory network connected to circRNAs that were highly expressed in GSC23 was developed. **C** These 10 mRNAs were negatively correlated with most infiltrating immune cells

This study discovered 144 mRNAs intimately linked to glioma patient prognosis (P < 0.001), among which 141 highly expressed mRNAs indicated a poor prognosis (hazard ratio > 1), revealing that these 141 mRNAs can be used as poor prognostic factors for glioma. Then, we constructed a PPI network and imported a PPI network diagram into Cytoscape3.8.2 software for visualization (Fig. 4E). Additionally, we identified the top 10 hub genes (AURKA, KIF20A, KIF2C, RAD51AP1, CENPN, ESPL1, HJURP, BIRC5, CDCA2 and ZWINT) (Fig. 4F). Through the GTEx and TCGA databases, this study revealed that the expression profile of 10 hub genes was significantly upregulated in glioma tissue compared with NC tissue, providing further evidence that such a gene network can have pivotal parts in glioma progression (Fig. 5A). Therefore, we hypothesize that highly expressed circRNAs in GSC23 are transported to glioma cells and have pivotal roles in glioma tumor development by regulating these 10 hub genes through miRNAs. Finally, we constructed a circRNA-miRNA-mRNA regulatory network with plausible links to circRNAs highly expressed in GSC23 (Fig. 5B).

Finally we constructed a circRNA-miRNA-mRNA network (including 8 circRNAs, 6 miRNAs and 10 mRNAs). In general we fitted RNA expression in glioma stem cells and glioma cells (circRNA up-regulated in GSC cells, miRNA down-regulated in glioma cells, mRNA up-regulated in glioma cells) and RNA target prediction together, to construct the circRNA-miRNA-mRNA network.

### Immunocorrelation analysis

Through immunocorrelation analysis, we found that these 10 mRNAs were negatively correlated with most of the infiltrating immune cells, suggesting that the high expression of these genes in glioma may inhibit tumor immunotherapy (Fig. 5C).

CircRNA-Serpine2 upregulates KIF20A expression by sponging miR-124–3p within gliomas.

**Fig. 6:**
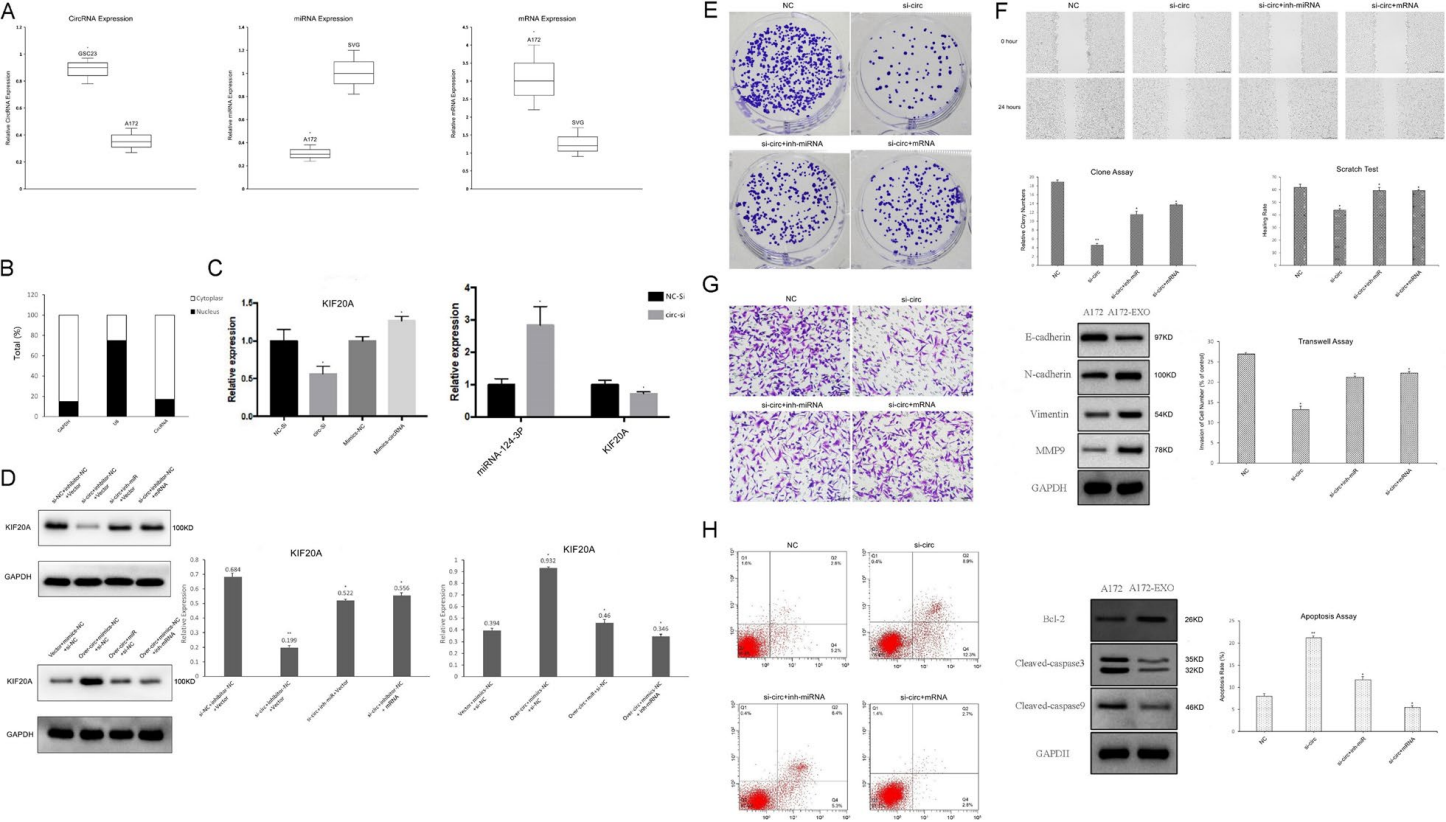
Circ-Serpine2 upregulates KIF20A expression, accelerates the proliferation, invasion, and migration of glioma cells, and inhibits apoptosis by sponging miR-124-3p. **A** Relative circ-Serpine2, miR-124-3p and KIF20A expression was detected by qRT-PCR. **B** The circRNA expression levels were assessed by qRT-PCR. GAPDH was used as a cytoplasmic marker, and U6 was used as a nuclear marker. **C** miR-124-3p and KIF20A expression was detected following the knockdown of circ-Serpine2. **D** KIF20A expression was detected by Western blot assays after transfection. **E** Colony formation assays were carried out with cotransfection of circ-Serpine2/miR-124-3p inhibitor or KIF20A plasmid in A172 cells. **F** A scratch test was used to measure the migration ability of A172 cells after transfection (0 h and 24 h). Pictures were captured under a light microscope at a magnification of 10 × 10. **G** Cell invasion assays were performed by cotransfecting A172 cells with circ-Serpine2/miR-124-3p inhibitor or KIF20A plasmid. The protein expression of invasion-related proteins was determined by Western blotting. **H** Apoptosis assays were performed by cotransfecting A172 cells with circ-Serpine2/miR-124-3p inhibitor or KIF20A plasmid. Protein expression of apoptosis-related proteins was determined through Western blotting. *P < 0.05 **P < 0.01

Compared with their levels in A172 cells, circ-Serpine2 was significantly upregulated in GSC23 cells, while compared with their levels in SVG cells, miR-124-3p was significantly downregulated and KIF20A was significantly upregulated in A172 cells (Fig. 6A). We investigated the subcellular localization of circ-Serpine2 to determine specific roles leading to glioma development. The results showed that circ-Serpine2 was localized predominantly to the cytoplasm of A172 cells, suggesting that it could regulate glioma physiology and pathology in posttranscriptional ways (Fig. 6B). MiR-124-3p upregulation and KIF20A downregulation were accompanied by the knockdown of circ-Serpine2. Overexpression of circ-Serpine2 upregulated KIF20A expression (Fig. 6C). Moreover, in our study, we added GSC23-secreted exosomes and GSC23-secreted exosomes deficient in circ-Serpine2 to A172 and U251 cells for coculture and co-transfected them with miR-124-3p/KIF20A inhibitor/mimics. The results indicated that circ-Serpine2 knockdown drastically reduced KIF20A levels in A172 cells, while inh-miR-124-3p inhibitor and KIF20A mimics could rescue this effect; furthermore circ-Serpine2 upregulation could promote KIF20A expression in U251 cells, whereas miR-124-3p mimics and KIF20A inhibitor could reverse this effect (Fig. 6D). In summary, such datasets provide evidence that circ-Serpine2 is a target of miR-124-3p as a miRNA sponge for eventual KIF20A upregulation.

### Circ-Serpine2 promotes glioma cell progression through the miR-124-3p/KIF20A nexus

In the functional verification section, GSC23-secreted exosomes were added to the normal control group, GSC23-secreted exosomes deficient in circ-Serpine2 were added to the si-circ group, GSC23-secreted exosomes deficient in circ-Serpine2 and miR-124-3P inhibitor were added to the si-circ + inh-miRNA group, and GSC23-secreted exosomes deficient in circ-Serpine2 and KIF20A mimics were added to the si-circ + miRNA group.

Clone forming assay dataset outcomes demonstrated that silencing circ-Serpine2 drastically reduced colony-forming capacity within A172 cells (Fig. 6E). The scratch assay suggested that circ-Serpine2 knockdown could thwart cell migration capacity (Fig. 6F). Regarding the Transwell assay, invasive cell abundance was drastically lowered in the si-circ-Serpine2 group. Moreover, in WB assay GSC23-secreted exosomes deficient in circ-Serpine2 were added in A172 group, while GSC23-secreted exosomes were added in A172 EXO group, the analysis demonstrated proteomic expression levels of N-cadherin, Vimentin, and MMP9 to be upregulated, while E-cadherin was decreased within A172 cells containing GSC23-secreted exosomes (Fig. 6G). Such outcomes indicated that circ-Serpine2 promoted glioma proliferation, together with migration/invasion in vitro.

To further validate this novel regulatory interaction between circ-Serpine2, miR-124-3p and KIF20A, this study also focused on the proliferative, migration and invasive capacities of A172 cells post-transfected with inh-miR-124-3p/KIF20A mimics. Outcomes suggested that miR-124-3p downregulation/KIF20A upregulation can recover si-circ-Serpine2-restricted proliferative, migration and invasive capacities (Fig. 6E–G). This evidence led to the conclusion that circ-Serpine2 promotes glioma cell proliferation, migration and invasion across the miR-124-3p/KIF20A nexus.

### Circ-Serpine2 thwarted apoptosis within gliomas via the miR-124-3p/KIF20A nexus

The apoptotic ratio was higher in the si-circ-Serpine2 group, while cotransfection with miR-124-3p inhibitor/KIF20A mimics reversed this effect. In WB assay GSC23-secreted exosomes deficient in circ-Serpine2 were added in A172 group, while GSC23-secreted exosomes were added in A172 EXO group, the Bcl-2 proteomic level was exacerbated while cleaved-caspase3/cleaved-caspase9 levels were reduced within cells exposed to GSC23 secreted exosomes (Fig. 6H). These results proved that circ-Serpine2 may thwart apoptosis within gliomas through miR-124-3p binding and upregulation of KIF20A.

### Circ-Serpine2 downregulation induced glioma growth regulation in vivo

**Fig. 7:**
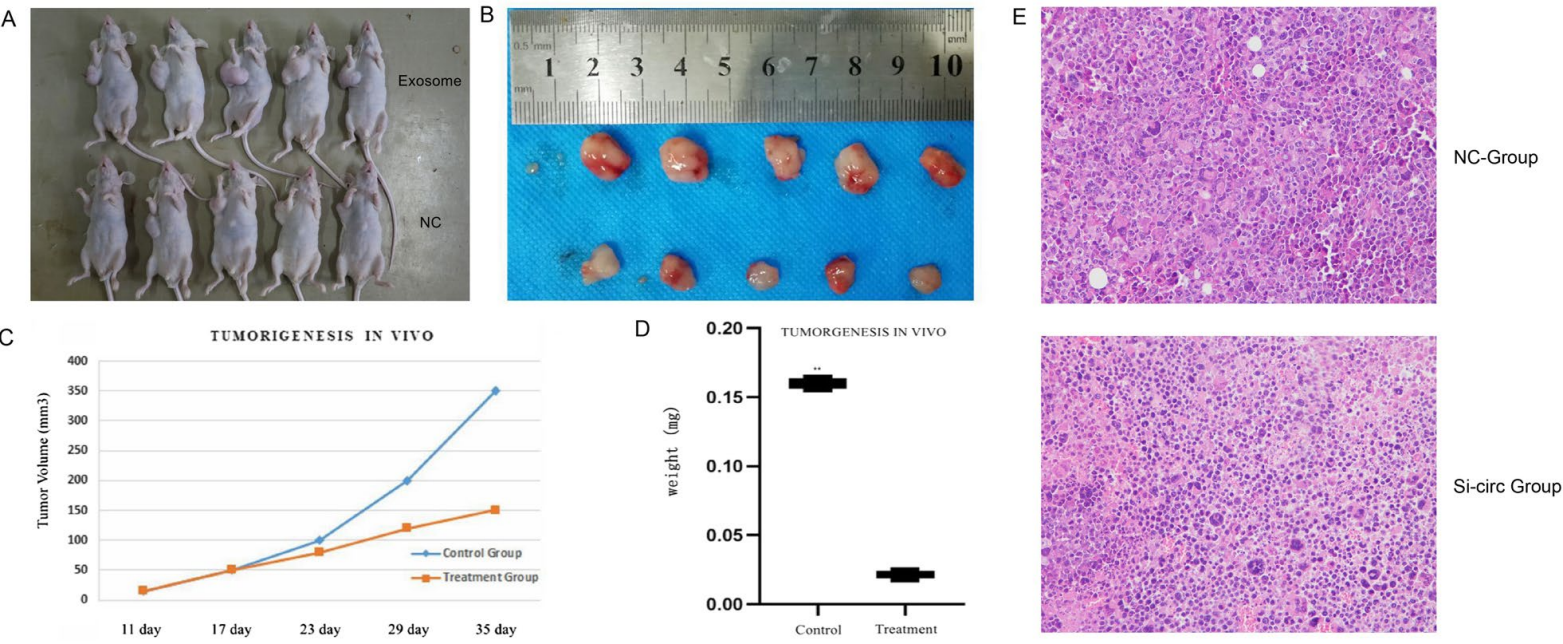
Circ-Serpine2 promotes glioma growth in vivo. **A** Tumor size in tumor-burdened rats was measured. **C** The growth rate was determined by measuring tumors at 6, 12, 18, 24 and 30 days after subcutaneous injection. **B**, **D** Tumors were harvested on day 35, and tumor weights were measured. **E** HE dye for subcutaneous tumor pathology sections. *P < 0.05 **P < 0.01

The results showed that in comparison to murines treated with normal A172 cells and GSC23 exosomes, tumor weight/volume readings were drastically reduced among tumor-carrying murines subjected to A172 and circ-Serpine2-deficient exosomes (Fig. 7A, B, D), with tumor weight analyses demonstrating statistical significance (P < 0.05). The time-dependent measurements reflected a considerable reduction in tumor development (Fig. 7C). HE staining demonstrated apoptotic activity within samples to be highly exacerbated within the si-circ-Serpine2 group (Fig. 7E). Further validating the capacity of circ-Serpine2 to accelerate glioma expansion in vivo.

## Discussion

High-grade gliomas are typically linked to weak prognosis and poor median survival times. An improved understanding of the mechanisms promoting glioma malignancy is needed to increase the efficacy of treatment [19–23], and accumulative evidence suggests that GSCs may participate glioma progression. Although an increasing number of biomarkers have been discovered, the relationship between GSCs and glioma progression is still unclear.

Previous studies have revealed that circRNAs, often termed miRNA sponges, possess differing miRNA response elements (MREs) that allow binding onto miRNAs, consequently downregulating cytoplasmic miRNAs and ultimately derepricing downstream targeted transcripts. In recent decades, various critical roles for circRNAs in tumor progression have been revealed. For example, Bian demonstrated that circRNA_103809 regulates colorectal cancer growth and migration [24]. Gao found that overexpression of hsa_circ_101280 upregulated the miR-124 target gene JAK2, which accelerated cellular proliferative properties [25]. CircZFR was identified to interact with C8orf4 by sponging miR-1261 in papillary thyroid carcinomas [26]. However, until now, the effects of circRNAs on the progression of glioma and their related mechanisms have rarely been reported. It has been postulated that glioma-derived exosomes may modulate target cells via the transfer of materials such as proteins, miRNAs, and noncoding RNAs [27–33]. This study focused on the possibility of GSC stemming exosomes being implicated in promoting glioma advancement through circRNA transport.

Until now, the precise functions of GSC-expressed circRNAs within glioma have remained uncertain. To establish whether GSCs expressing circRNAs could act as ceRNAs within glioma, this study initially conducted circRNA sequencing and used microarray data and the limma package in R to perform a differential analysis between GSC23 and differentiated GSC23 samples. A circRNA-miRNA-mRNA regulatory network was consequently developed, founded upon predictive models in biology, and prepared a model PPI network for DEm-RNAs. In addition, circRNA-miRNA-hub and miRNA-mRNA gene subnetworks were also developed and found to regulate modules flagged within the circRNA-miRNA-mRNA network.

Through functional enrichment experiments, mRNA functional exploration, CGGA data, Kaplan–Meier survival analysis, PPI network and immune-related research, we finally identified eight circRNAs (hsa_circ_001588, hsa_circ_002006, hsa_circ_000898, hsa_circ_001484, hsa_circ_001101, hsa_circ_001501, hsa_circ_000735, hsa_circ_001714), six miRNAs (hsa-miR-124-3p, hsa-miR-129-1-3p, hsa-miR-346, hsa-miR-485-5p, hsa-miR-495-3p, hsa-miR-760) and ten mRNAs (AURKA, KIF20A, KIF2C, RAD51AP1, CENPN, PLES1, BIRP, BIRC5, hsa-ZWINT) that can have pivotal parts within KIF20A progression. This investigation identified that KIF20A showed significantly high expression in glioma tissues, which suggested that this hub gene could have an essential place for exacerbating glioma expansion. Additionally, its expression was negatively correlated with infiltrating immune cells, which indicated that its high expression may inhibit tumor immunotherapy.

Previous studies have explored the regulatory effects of related circRNAs, miRNAs, and mRNAs on tumor occurrence and development. For example, Valentina reported that Serpine2 influences glioma cell migrative/invasive properties by regulating uPA and MMP-9/2 levels [34]. Vaillant analysis showed that Serpine2 boosts the malignant progression of PNLs to medulloblastomas [35]. Wu reported that miR-124-3p could impede malignant phenotypic manifestations within gliomas through inhibition of EphA2 [36]. Lang found drastic miR-124 downregulation in GSCs compared with NSCs. Liu reported that miR-124-3p promoted apoptosis and growth inhibition in GSCs [37]. Zhou reported that KIF20A was a hub gene and mainly played a part within the cell cycle and p53 signaling activities [38]. Saito reported that KIF20A was significantly upregulated, particularly within glioma tissues, throughout the process of cellular mitotic activities. In silico analyses identified KIF20A to be upregulated within gliomas, according to tumor grade, with glioma patients having upregulated KIF20A experiencing poor prognoses. Downregulation of KIF20A decreased cellular proliferative properties within gliomas through lack of cytokinesis binucleated cell development. Moreover, KIF20A blockade led to enhanced apoptotic activity within SF126 glioma cells [39].

Through circRNA sequencing and differential analysis, we found that hsa_circ_001588 (Serpine2) was highly upregulated in GSC23 compared to glioma cells, miR-124-3p was severely downregulated in glioma cells, and KIF20A was significantly upregulated in gliomas. By constructing a GSC-specifically expressed circRNA-miRNA-mRNA regulatory network and performing mRNA functional exploration and immune-correlation analysis, we further discovered that circ-Serpine2 may competitively inhibit miR-124-3p expression and eventually influence KIF20A expression, which could promote glioma malignant progression and suppress glioma immunotherapy.

In our signaling pathway regulation experiment, we found that the silencing of circ-Serpine2 could increase miR-124-3p expression and decrease KIF20A expression in glioma cells. Subsequently, Western blotting demonstrated that circ-Serpine2 knockdown could downregulate KIF20A, with miR-124-3p cosilencing and KIF20A mRNA providing restoration of such reduced activity. In contrast, overexpression of circ-Serpine2 elevated KIF20A levels, and cotransfection with either miR-124-3p mimics or si-KIF20A reversed this effect. Taken together, these data indicated that circ-Serpine2 upregulates the KIF20A downstream signaling pathways by sponging miR-124-3p.

Functional analyses revealed that circ-Serpine2 knockdown reduced glioma proliferative, migrative and invasive properties in vitro. Such a circ-Serpine2-induced reduction could be counteracted by downregulating miR-124-3p and upregulating KIF20A expression. Reducing circ-Serpine2 expression also promoted glioma apoptosis and was rescued through a miR-124-3p inhibitor or KIF20A mimics.

The animal model analysis showed that circ-Serpine2 downregulation resulted in reduced tumor volumes and weights. Examination of HE-stained tumor sections showed increased numbers of apoptotic cells after circ-Serpine2 silencing. These findings indicated that circ-Serpine2 can promote tumor growth in vivo.

In conclusion, in this research, we identified differentially expressed circRNAs and constructed a circRNA-miRNA-mRNA regulatory network that putatively participates in glioma cell regulation. Then, we demonstrated that GSC23 cell exosome-secured circ-Serpine2 can exacerbate KIF20A expression by sponging miR-124-3p, thus promoting glioma proliferation, migration and invasion in vitro and inhibiting glioma apoptosis in vitro. In addition, our research indicated that circ-Serpine2 inhibited glioma apoptosis and promoted tumor expansion in vivo. Such evidence indicated that circ-Serpine2 modulates glioma malignancy through the miR-124-3p/KIF20A nexus. Based on these experimental results, we could develop novel predictive/prognostic biomarkers and drug target-based therapeutic strategies in glioma clinical management.

## Data Availability

Once the article is accepted by the Journal, our circRNA sequencing results will be uploaded to NCBI SRA database. The datasets analyzed during the current study are available from the corresponding author on reasonable request.
